# Supplementary material for: Resolving the full spectrum of human genome variation using Linked-Reads
Source: Genome Res. 2019 Apr;29(4):635–45. doi: 10.1101/gr.234443.118 (PMC6442396; doi:10.1101/gr.234443.118)
Supplement: Supplemental Material [file supp_gr.234443.118_Supplemental_longranger-2.2.zip › longranger-2.2.tar.bz2!/lr2.2-public/lib/stan/cmdstan/stan/doc/api/html/base__adaptation_8hpp_source.html]

Stan: src/stan/mcmc/base\_adaptation.hpp Source File


|  |  |
| --- | --- |
|  | Stan  2.5.0  probability, sampling & optimization |


- Main Page
- Related Pages
- Namespaces
- Classes
- Files

- File List
- File Members

All Classes Namespaces Files Functions Variables Typedefs Enumerations Enumerator Friends Macros Pages

- src
- stan
- mcmc

base\_adaptation.hpp

Go to the documentation of this file.

1 #ifndef STAN\_\_MCMC\_\_BASE\_\_ADAPTATION\_\_BETA

2 #define STAN\_\_MCMC\_\_BASE\_\_ADAPTATION\_\_BETA

3

4 namespace stan {

5

6  namespace mcmc {

7

8  class base\_adaptation {

9

10  public:

11

12  virtual void restart() {};

13

14  };

15

16  } // mcmc

17

18 } // stan

19

20 #endif

stan::mcmc::base\_adaptation::restart

virtual void restart()

**Definition:** base\_adaptation.hpp:12

stan::mcmc::base\_adaptation

**Definition:** base\_adaptation.hpp:8


---

[ Stan Home Page ]

*© 2011–2014,
Stan Development Team.*
